# Evaluation of early immune response in multitrauma patients admitted in ICU: the effect of age

**DOI:** 10.1186/2197-425X-3-S1-A372

**Published:** 2015-10-01

**Authors:** D Markopoulou, K Venetsanou, V Kaldis, D Chatzilia, D Chroni, S Stratouli, I Alamanos

**Affiliations:** 2nd ICU, KAT General Hospital Kifisia, Athens, Greece; Research Unit, KAT General Hospital Kifisia, Athens, Greece

## Introduction

It is known the negative effect of urgent clinical incidents on the immunological status of patients. Several clinical and demographic data, including age are related to it.

## Objectives

The aim of the study is the evaluation of early immune response in multitrauma patients on admission in ICU according to patient's age.

## Methods

Thirty two multitrauma patients issued in ICU included in the study classified into two groups according to their age, (I) adults ≤65 (N = 14) and (II) elderly >65 (N = 18) years old. Ten milliliters of peripheral blood collected from each patient on admisssion, divided into two tubes with/without anticoagulant. Whole blood samples diluted 1:10 with RPMI 1X, incubated with /without 500pg/ml LPS at 37°C for 4H. Serum and cell culture supernatants (CCSPs) isolated by centrifugation and stored at -70°C. Tumour necrosis factor alpha (TNF-a) and interleukins 6, 8, 10 (IL-6, IL-, IL-10) measured in serum and CCSPs with ELISA. Statistical analysis was performed with Graphpad 5.0. Data are presented as Median +/- IQRs; a P value < 0.05 accepted as significant.

## Results

Serum TNF-a was significantly higher in elderly (P < 0.01), while IL-6, IL-8 and IL-10 had no significant differences between groups. Opposite, ex vivo cytokines release was significantly lower in elderly (I)compare to adults (II), TNF-a (P < 0.05), IL-6 (P < 0.01) and IL-8 (P < 0.01) but not IL-10 (P > 0.05).

## Conclusions

The effect of age seems to be critical on the immune response of severe trauma patients at the early state of the incident.

**Figure 1:**
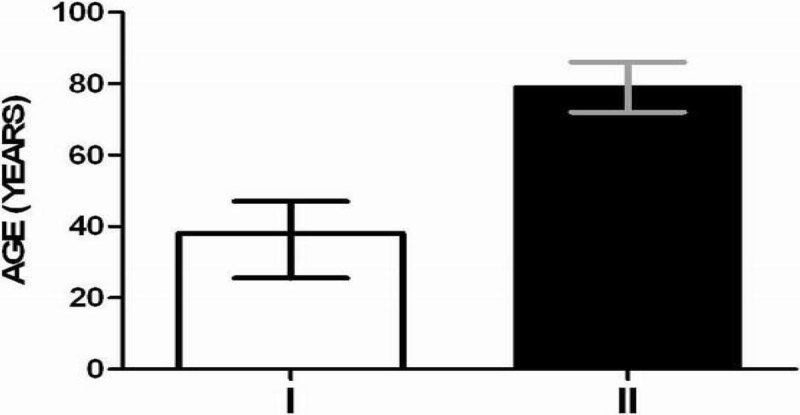
**Patient's age in groups (I) adults ≤65 and (II) elderly >65.**

**Figure 2:**
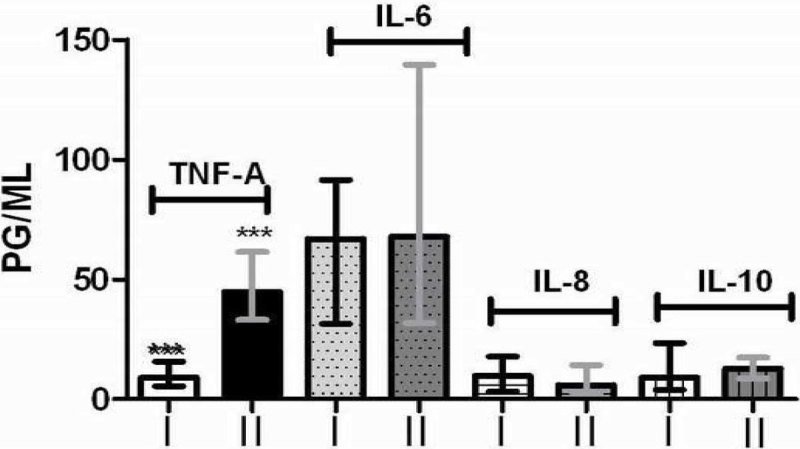
Serum cytokines levels in groups I and II. ***P < 0.001.

**Figure 3:**
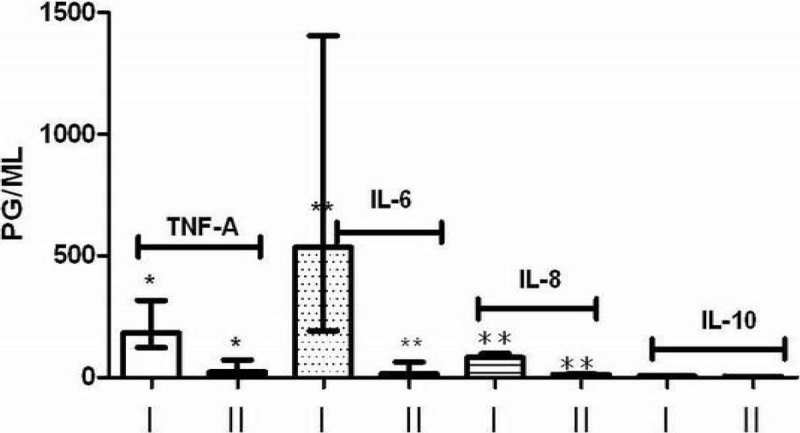
Ex vivo LPS cytokines release in groups I and II, after baseline removal. *P < 0.0, **P < 0.01.
